# Microbial Synthesis of Alka(e)nes

**DOI:** 10.3389/fbioe.2013.00010

**Published:** 2013-10-16

**Authors:** Weihua Wang, Xuefeng Lu

**Affiliations:** ^1^Key Laboratory of Biofuels, Shandong Provincial Key Laboratory of Energy Genetics, Qingdao Institute of Bioenergy and Bioprocess Technology, Chinese Academy of Sciences, Qingdao, China

**Keywords:** alka(e)nes, microbial synthesis, synthetic biology, metabolic pathways, combinatorial biosynthesis

## Abstract

Alka(e)nes are the predominant constituents of gasoline, diesel, and jet fuels. They can be produced naturally by a wide range of microorganisms. Bio-alka(e)nes can be used as drop-in biofuels. To date, five microbial pathways that convert free fatty acids or fatty acid derivatives into alka(e)nes have been identified or reconstituted. The discoveries open a door to achieve microbial production of alka(e)nes with high efficiency. The modules derived from these alka(e)ne biosynthetic pathways can be assembled as biological parts and synthetic biology strategies can be employed to optimize the metabolic pathways and improve alka(e)ne production.

## Introduction

Biofuel production from renewable sources is becoming more and more attractive because of the rapidly increasing consumption and irreversibly diminishing reserves of petroleum. Advanced biofuels produced from biological systems should be chemically similar to petroleum-based fuels. It is necessary to develop advanced biofuels besides widely used bioethanol and biodiesel (Keasling and Chou, 2008). Alka(e)nes of defined chain lengths possess higher energy density, hydrophobic property, and compatibility with existing liquid fuel infrastructure including fuel engines, refinery equipment, and transportation pipelines, and they can serve as better alternatives to petroleum-based fuels (Peralta-Yahya et al., 2012).

Several emerging researches on alka(e)ne biosynthesis were reported in 1940s and 1950s from sulfate-reducing bacteria and some marine bacteria (Zobell, 1945; Stone and Zobell, 1952). By the 1960s, investigations of microbial production of alka(e)nes were accelerated with the development of analytical techniques such as nuclear magnetic resonance (NMR), gas chromatography (GC), and mass spectrometry (MS). Alka(e)ne production was reported in a diversity of microorganisms including bacteria (Jones, 1969; Tornabene et al., 1970), algae (Gelpi et al., 1968), yeasts (Merdinge and Frye, 1966), and fungi (Oro et al., 1966; Walker and Cooney, 1973).

It was reported that a variety of alka(e)nes including long-chain and branched molecules were produced in cyanobacteria and heptadecane is the most abundant since 1960s (Han et al., 1969; Winters et al., 1969). In particular, cyanobacteria are unique in their ability to produce 7- and 8-methylheptadecanes in a ratio of 1:1, which was shown to be derived from 11-octadecenoic acid by the addition of methyl-^14^C group of methionine to a double bond (Fehler and Light, 1970). The alka(e)ne profile of algae was dominated by the odd-numbered n-alkanes, typically at C_15_, C_17_, or C_21_. *Botryococcus braunii*, a green alga, contains exceptionally high content of alka(e)nes (Brown et al., 1969; Banerjee et al., 2002). Yeasts are able to synthesize a wide range of alka(e)nes including not only n-alkanes but also unsaturated and branched components (Merdinge and Frye, 1966), whereas in fungi, long-chain alka(e)nes are predominant (Merdinge and Devine, 1965).

Mechanisms of alka(e)ne biosynthesis have been investigated biochemically long before availability of genetic information. Two alka(e)ne biosynthetic mechanisms were studied extensively. In “elongation–decarboxylation,” an acyl-coenzyme A (CoA) is elongated by the circular addition of a two-carbon unit derived from malonyl-CoA to an acyl-CoA with the subsequent decarboxylation to form an odd-chain alka(e)ne (Dennis and Kolattukudy, 1991, 1992). In “head-to-head condensation,” two fatty acid derivatives are conjugated to carboxylic acid by Claisen condensation, followed by decarboxylation and decarbonylation to form an odd-chain alkene (Albro and Dittmer, 1969b, 1970; Bird and Lynch, 1974). This review focuses on the diversified biosynthetic pathways of alka(e)nes.

## Identified Biosynthetic Pathways of Alka(e)nes

**Figure 1:**
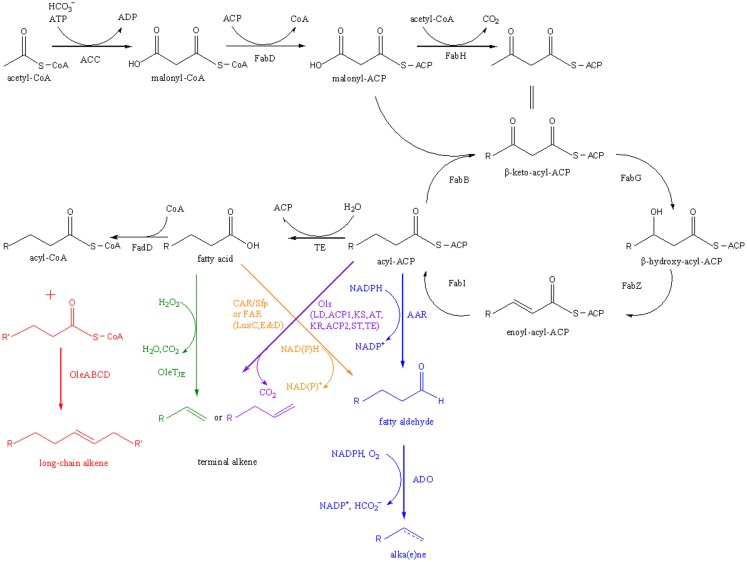
**Pathways for fatty acid-based alka(e)ne biosynthesis**. Five alka(e)ne biosynthetic pathways are shown in different colors. ACC, acetyl-CoA carboxylase; FabD, malonyl-CoA:ACP transacylase; FabH, β-keto-acyl-ACP synthase III; FabB, β-keto-acyl-ACP synthase I; FabG, β-keto-acyl-ACP reductase; FabZ, β-hydroxyacyl-ACP dehydratase; FabI, enoyl-acyl-ACP reductase; TE, thioesterase; FadD, acyl-CoA synthase; AAR, acyl-ACP reductase; ADO, aldehyde-deformylating oxygenase; OleABCD, a four protein families for long-chain olefin biosynthesis; OleT_JE_, a cytochrome P450 enzyme that reduces fatty acids to alkenes; CAR, carboxylic acid reductase; Sfp, A phosphopantetheinyl transferase; FAR, fatty acid reductase; Ols, a type I polyketide synthases for α-olefin biosynthesis.

Five microbial pathways that convert free fatty acids (FFAs) or fatty acid derivatives into alka(e)nes have been identified or reconstituted (Figure 1).

### Pathway 1: AAR-ADO

A subtractive genome analysis was employed by Schirmer et al. (2010) to identify cyanobacteria alkane biosynthetic pathway genes. No alkane biosynthesis was observed in *Synechococcus* sp. PCC7002, so 10 other alkane-producing cyanobacteria strains with sequenced genome and *Synechococcus* sp. PCC7002 were investigated. A 40% sequence identity was used to subtract the *Synechococcus* sp. PCC7002 genome by the other 10 genomes to cut off orthologs. After excluding 15 genes with unrelated functions, two candidate enzymes for alkane biosynthesis were identified and verified. Acyl–acyl carrier protein (ACP) can be reduced to aldehyde by an acyl-ACP reductase (AAR, EC 1.2.1.50), and then aldehyde can be converted to alkane or alkene with formation of carbon monoxide as a co-product by an aldehyde decarbonylase (ADC, EC 4.1.99.5) (Schirmer et al., 2010).

In the following research, formate $\left({\text{HCO}}_{2}^{-}\right)$ rather than CO was identified as the stoichiometric co-product of the reaction converting fatty aldehyde to alkane or alkene by *Nostoc punctiforme* PCC73102 ADC (NpunR1711) overexpressed in *Escherichia coli*. Radiolabeled experiments indicated that the aldehyde hydrogen was retained in the formate and the hydrogen in the nascent methyl group derives at least partially from solvent (H_2_O) (Warui et al., 2011).

Conversion of fatty aldehydes to alka(e)nes and formate seems to be redox-neutral and formally hydrolytic, while the reaction is actually a novel redox oxygenation process. Cyanobacterial ADC is a non-heme di-iron oxygenase, which requires a reducing system and O_2_ to catalyze the conversion of fatty aldehydes to alka(e)nes and formate (Li et al., 2011). Isotopetracer experiments showed that O_2_ was involved in formation of a metal-bound peroxide nucleophile to attack the aldehyde and one oxygen atom is incorporated from O_2_ into formate. The oxygenative nature of the reaction implies that the enzyme ADC should be redesignated as aldehyde-deformylating oxygenase (ADO) (Li et al., 2012). It was reported that ADO activity is reversibly inhibited by H_2_O_2_ originating from poor coupling of reductant consumption with alka(e)ne formation, and the inhibition can be relieved by a fusion catalase (Andre et al., 2013).

*In vitro* activity of the cyanobacterial ADO requires a bio-reducing system (NADPH, ferredoxin, and ferredoxin reductase) or a chemical reducing system (NADH and phenazine methosulfate). The potential endogenous reducing system including ferredoxin and ferredoxin reductase in *Synechococcus elongatus* PCC7942 supported greater ADO activity and produced less H_2_O_2_ than the spinach reducing system and chemical ones (Zhang et al., 2013).

Alkane biosynthetic genes from various cyanobacteria were heterologously expressed in *E. coli* and *Synechococcus* sp. PCC 7002. A variety of C13-C17 alka(e)nes were produced in *E. coli* and the highest alka(e)ne titers were over 300 mg/L. In *Synechococcus* sp. PCC 7002, the alka(e)ne content reached 5% of cell dry weight (DW) (Reppas et al., 2010; Schirmer et al., 2010).

Overexpression of both AAR and ADO from several cyanobacteria strains in *Synechocystis* sp. PCC6803 led to a doubled alka(e)ne production. Redirecting the carbon flux to acyl-ACP and overexpression of alkane biosynthetic genes simultaneously can significantly improve alka(e)ne production in engineered *Synechocystis* strains. Alka(e)ne content in a *Synechocystis* mutant overexpressing alkane biosynthetic genes in both *slr0168* and *slr1556* gene loci was 1.3% of DW, which was enhanced by 8.3 times compared with wild-type strain (0.14% of cell DW) (Wang et al., 2013).

### Pathway 2: Head-to-head

A head-to-head condensation of fatty acid derivatives to generate long-chain olefins has been reported for more than 40 years (Albro and Dittmer, 1969a), while the genetic information of olefin biosynthesis were not elucidated until 2010. A three-gene cluster from *Micrococcus luteus* (Mlut_13230–13250) was identified as olefin biosynthetic genes (*ole*) by bioinformatics and biochemical analysis. Heterologous expression of the *ole* cluster from *M*. *luteus* in fatty acid-overproducing *E. coli* resulted in about 40 μg/L long-chain alkenes (Beller et al., 2010).

The head-to-head condensation reaction requires four protein families (OleABCD). The *oleB* and *oleC* genes are fused and encode a multi-domain protein (for example, Mlut_13230) in a variety of bacteria. Putative *ole* genes were identified in 69 out of 3558 genomes analyzed by sequence alignments and gene context analysis (Sukovich et al., 2010a). The OleABCD protein families were defined within thiolase, α/β-hydrolase, AMP-dependent ligase/synthetase, and short-chain dehydrogenase superfamilies, respectively.

In-depth studies on head-to-head olefin biosynthesis have focused on OleA. OleA catalyzes the first committed step in the olefin biosynthetic pathway and plays a critical role in determining product structure. A Claisen condensation of fatty acid derivatives is catalyzed by OleA to generate a β-ketoacid that can decarboxylate spontaneously to generate ketones (Frias et al., 2011). The production of olefins required the presence of OleC and OleD, which catalyze further reactions with the β-ketoacid intermediate generated by OleA (Sukovich et al., 2010b; Frias et al., 2011). The conservation of *oleB* in the *ole* gene clusters suggests that it does play a role in olefin biosynthesis, but no mechanism or defined role of OleB has been reported (Sukovich et al., 2010a).

To understand the substrate positioning in the context of enzyme turnover, the crystal structures of unbound OleA from *Xanthomonas campestris*, OleA-cerulenin, and xenon-derivatized OleA-cerulenin cocrystals were determined. The active site of OleA contains a cysteine (Cys143) and a glutamic (Glu117β) residue. An additional long-chain alkyl binding channel required for OleA catalysis was assigned (Goblirsch et al., 2012).

### Pathway 3: OleT_JE_

Terminal olefins (1-alkenes) synthesis is widespread in bacteria of the genus *Jeotgalicoccus*. The key enzyme to convert FFAs to terminal olefins in *Jeotgalicoccus* sp. ATCC 8456 was isolated by purification from cell extract, and its encoding gene was identified from a draft genome sequence of *Jeotgalicoccus* sp. ATCC 8456 using reverse genetics. The enzyme was named OleT_JE_ (Rude et al., 2011).

Heterologous expression of *oleT*_JE_ conferred terminal olefin production to *E. coli.* However, the olefin titer in *E. coli* expressing *oleT*_JE_ was not reported. The fatty acid decarboxylase OleT_JE_ is a P450 peroxygenase from the cyp152 family, which directly decarboxylate long-chain FFAs (C_16_–C_20_) to α-olefin using H_2_O_2_ as its main source of electrons. It is indicated that OleT_JE_ also includes fatty acid hydroxylase activity and His85 in OleT_JE_ plays an important role in catalysis (Rude et al., 2011).

### Pathway 4: Ols

A marine cyanobacterium *Synechococcus* sp. PCC 7002 can synthesize 1 nonadecene and 1,14 nonadecadiene with terminal double bond. The *curM*-encoding enzyme is responsible for forming the terminal double bond of Curacin A in a marine cyanobacterium *Lyngbya majuscula* (Gu et al., 2009). Homologs to CurM were searched in *Synechococcus* sp. PCC 7002 by NCBI basic local alignment search tool (BLAST) and an open reading frame (ORF) encoding a protein with 45% amino acid sequence identity to CurM was identified. The ORF was named *ols* (olefin synthase) (Mendez-Perez et al., 2011).

The *ols* gene encodes a large multi-domain type I polyketide synthases (PKS). Acyl-ACP is loaded to the ACP1 domain by the loading domain (LD), then two carbons from malonyl-CoA are added to the acyl-substrate by the central extension module including ketosynthase (KS), acyltransferase (AT), ketoreductase (KR), and ACP2 domain and the β-keto group is reduced to a β-hydroxyl. The sulfotransferase (ST) domain activates the β-hydroxyl group via sulfation. Subsequent dehydration and decarboxylation reactions could be catalyzed by the C-terminal thioesterase (TE) domain (Mendez-Perez et al., 2011).

The native promoter of the *ols* gene was replaced with the promoter of *psbA* in *Amaranthus hybridus*. Production of 1 nonadecene and 1,14 nonadecadiene were increased two- and five-fold respectively in the promoter replacement mutants. The highest observed olefin titer was about 4.2 μg/ml/OD_730_. A 2.2-fold increase in mRNA level was observed in the mutant relative to the wild-type strain (Mendez-Perez et al., 2011).

### Pathway 5: CAR/FAR-ADO

Recently, artificial alkane biosynthetic pathways were designed and implemented for the production of alka(e)nes in *E. coli*. The carboxylic acid reductase (CAR) from *Mycobacterium marinum* together with the phosphopantetheinyl transferase Sfp from *Bacillus subtilis* (Quadri et al., 1998) and fatty acid reductase (FAR) complex encoded by the genes *luxC, luxE*, and *luxD* from *Photorhabdus luminescens* can catalyze formation of fatty aldehydes from FFAs (Akhtar et al., 2013; Howard et al., 2013). Coupled with ADO from cyanobacteria, fatty aldehydes can be converted further to alka(e)nes.

Genetic parts such as certain thioesterase, lipase, the branched-chain α-ketoacid dehydrogenase complex and β-keto-acyl-ACP synthase III from *B. subtilis* can be introduced to *E. coli* for targeted manipulation of the fatty acid pool to synthesize straight-chain or branched (*iso*-) alkanes (Howard et al., 2013). However, alka(e)ne titers from these artificial alkane biosynthetic pathways were only ∼2–5 mg/L. It is proposed that a much more efficient competing pathway diverting aldehyde to fatty alcohol by an *E. coli* native fatty aldehyde reductases leads to the low alka(e)ne productivity (Akhtar et al., 2013).

## Future Trends

The five microbial alka(e)ne biosynthetic pathways are all derived from fatty acid metabolism. To date, extensive genetic and biochemical studies were performed on “AAR-ADO” and “Head-to-Head” pathways. Linear and branched alkanes can be produced in “AAR-ADO” and “CAR/FAR-ADO” pathways. The unique P450 enzyme OleT_JE_ can directly convert long-chain FFAs (C_16_–C_20_) to α-olefin, which is a short cut to alkenes. The gap between current biosynthetic efficiency and commercial alka(e)ne production remains huge (Table 1).

**Table 1:** **Production level reported for the five alka(e)ne biosynthetic pathways**.

Pathway	Alka(e)ne	Host strain	Titer (reference)
AAR-ADO	Heptadecane and heptadecene	*Synechocystis* sp. PCC6803	1.3% of DW^a^ (Wang et al., 2013)
	Tridecane, pentadecene, pentadecane, and heptadecene	*E. coli*	300 mg/L (Schirmer et al., 2010)
	Pentadecane and heptadecane	*Synechococcus* sp. PCC 7002	5% of DW^a^ (Reppas et al., 2010 US7794969-B1)
Head-to-Head	27:3, 27:2, 29:3, and 29:2 alkene	*E. coli*	40 μg/L (Beller et al., 2010)
OleT_JE_	1,10-Heptadecadiene and 1-pentadecene	*E. coli*	NR^b^
Ols	1 Nonadecene and 1,14 nonadecadiene	*Synechococcus* sp. PCC 7002	4.2 μg/ml/OD_730_ (Mendez-Perez et al., 2011)
CAR/FAR-ADO	Linear undecane, tridecane, tridecene, pentadecane, pentadecene, hexadecane, hexadecene, heptadecane, heptadecene, and branched tridecane and pentadecane	*E. coli*	∼2–5 mg/L (Akhtar et al., 2013; Howard et al., 2013)

^a^ DW, cell dry weight.

^b^ NR, not reported

Diversified alka(e)ne biosynthetic pathways provide a rich genetic source to design and engineer microbes through synthetic biology for microbial production of alka(e)nes. Combining the synthetic modules of different alka(e)ne biosynthetic pathways can divert flux to target products. First, enzymology studies of key enzymes will facilitate rational enzymatic engineering to enhance the catalytic efficiency and alter substrate specificities. Directed evolution can be employed to screen libraries of enzyme variants for the desired characteristics. Second, deletion of competing pathways in the engineered strains can increase flux toward production of alka(e)ne. Heterologous genes/pathways can also be introduced to some platform microorganism to avoid the native post-translational regulation. Third, combinatorial biosynthesis strategies can be applied in type I PKS Ols. The existing domain of Ols could be replaced with modules from other type I PKS pathways to modify the structure and improve the properties and yield of alkenes. Domains of Ols can also be introduced to other alka(e)ne biosynthetic pathways to improve the efficiency of alka(e)ne biosynthesis. Last but not least, precise and dynamic control of mRNA and protein levels by genetic parts would enable adjustments for redox balance, ATP usage, and cofactor requirements according to the metabolic flux. In a conclusion tremendous efforts need to be paid toward genetic modifications of biosynthetic pathways of alka(e)nes for significant improvement of alka(e)nes production titers. Both synthetic biology and metabolic engineering approaches are powerful tools for optimizing alka(e)ne biosynthetic pathways.

## Conflict of Interest Statement

The authors declare that the research was conducted in the absence of any commercial or financial relationships that could be construed as a potential conflict of interest.
